# The contribution of microbial biotechnology to economic growth and employment creation

**DOI:** 10.1111/1751-7915.12845

**Published:** 2017-09-04

**Authors:** Kenneth Timmis, Victor de Lorenzo, Willy Verstraete, Juan Luis Ramos, Antoine Danchin, Harald Brüssow, Brajesh K. Singh, James Kenneth Timmis

**Affiliations:** ^1^ Institute of Microbiology Technical University of Braunschweig Braunschweig Germany; ^2^ Centro Nacional de Biotecnología Madrid Spain; ^3^ Center for Microbial Ecology and Technology (CMET) Ghent University Ghent Belgium; ^4^ Estacion Experimental del Zaidin Granada Spain; ^5^ ICAN CHU Pitié‐Salpêtrière Paris France; ^6^ Chaumeny La Tour de Peilz Switzerland; ^7^ Hawkesbury Institute for the Environment Western Sydney University Penrith SA Australia; ^8^ Student MSc Health Policy Department of Surgery and Cancer Imperial College London UK

## Abstract

Our communication discusses the profound impact of bio‐based economies – in particular microbial biotechnologies – on SDG 8: Promote sustained, inclusive and sustainable economic growth, full and productive employment and decent work for all. A bio‐based economy provides significant potential for improving labour supply, education and investment, and thereby for substantially increasing the demographic dividend. This, in turn, improves the sustainable development of economies.

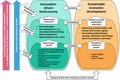

## Sustainable economic growth requires innovation and adequate human capital and employment

In the 2030 Agenda for Sustainable Development (see e.g. United Nations, 2015a,b), which places *people, planet, prosperity, peace and partnership* at the centre of an action plan encapsulated in the Sustainable Development Goals, economic growth, enterprise creation and employment opportunities represent a key building block that is articulated in SDG 8: *Promote sustained, inclusive and sustainable economic growth, full and productive employment and decent work for all*.

Traditional primary and secondary business sectors have contributed significantly to economic growth in particular in high‐ and middle‐income countries; however, these sectors are primarily based on redistribution/transformation, i.e. mining, harvesting, refining, processing, utilizing and disposing of finite (e.g. ore, oil) or temporally limited (e.g. crops) resources. Even a purely anthropocentric view of the planet is constrained by the fact that it and its inhabitants are subject to basic ecological rules (the food/energy pyramid, functional changes in the planetary ecological network induce compensatory reactions elsewhere in the network, etc.), finite resources and ecological consequences of environmental abuse. Thus, the notion that perpetual economic growth of traditional business sectors is possible and sustainable is unrealistic. The central task is thus to focus strategic economic development investment in new business growth sectors that most effectively and sustainably *inter alia* increase productivity, streamline consumption and scale down waste, in order to create a healthy economic growth trajectory within the global context. This will necessitate implementation of strategic policies designed to drive development of innovative solutions, which efficiently maximize the utility of local and regional advantages, incentivize diversification, establish centres of excellence, foster transnational public and private partnerships, attract investment, etc., to sustainably increase domestic and international value and, in turn, grow economies. Here we (i) argue that biotechnology and, in particular, microbial biotechnology offer huge potential to sustainably drive enterprise, employment and expertise creation and (ii) provide some examples of challenges and solutions, that may guide policy makers in the formulation and implementation of relevant policies that will facilitate economic growth.

## Population growth, demographic transition and the *demographic dividend*

Without doubt, one major factor requiring particular consideration in sustainable development strategies is the rate at which the global community has been growing and is projected to grow in the foreseeable future. The dramatic growth rates witnessed since the middle of the 20th century, most prominently in developing regions, are *inter alia* due to significantly improved public health measures, in particular measures to prevent infectious diseases and reduce malnutrition, which predominantly affect children. Such measures have achieved substantial reductions in infant mortality and increases in life expectancy, improved overall health and resulted in important shifts in age distributions in low‐ and middle‐income countries (lower average ages): *the demographic transition*. As the risk of infant mortality declines, parents tend to have fewer children; as urbanization, vocational prospects and salaries increase, the demand for a more highly skilled workforce grows, and the relative benefit of basic skills, such as farming, declines; as, in turn, employment prospects and return on investment in higher skills improve, parents tend to invest more in the education of their offspring to improve their chances in the labour market. This leads to successive generations of increasingly highly trained individuals which, over time, substantially increase the proportion of economically active individuals in a population and, in turn, significantly improve the potential for economic growth and development: *the demographic dividend*. However, exploitation of this economic growth potential is highly dependent on utilization of a window of opportunity (circa one century) and, importantly, a favourable and responsive policy environment, principally regarding the adequate support of the new *labour supply* and the increases in *savings* and *human capital* (see e.g. Bloom *et al*., 2003; Bloom, 2015).

Maximizing the potential of this new labour supply, and thereby exploiting the momentum to grow the economy, requires policies that focus on sustainably ensuring adequate job opportunities for the growing and increasingly skilled workforce. Due to increased productivity and hence earnings by public and private organizations, and individual households, capital savings (pensions, tax revenues, private equity, etc.) become available. Policies need to adequately regulate and increase trust in capital markets, and incentivize national and international trade, and, importantly, diversified individual and aggregate domestic (and foreign direct) investment, in particular in growth sectors and research and development. Improvements in education and health, which are mutually re‐enforcing and offer substantial return on investment for individuals and society as a whole, are central for economic growth. Therefore, policies should focus on creating and appropriately fostering an environment to sustainably increase social and human capital with a view to consolidating current, and paving the way for improved, economic growth trajectories (see e.g. Bloom *et al*., 2003; McTaggart *et al*., 2012). In addition, during transition from low‐ to middle‐ to high‐income settings, policy makers must be aware of and responsive to the changing determinants of economic growth pertinent to their setting (e.g. workforce specialization) and adapt policies accordingly to most effectively sustain growth momentum and avoid productivity stagnation, sometimes also referred to as the *‘*low‐growth trap’ (see e.g. Agénor and Canuto, 2015; Bulman *et al*., 2017; Engel and Taglioni, 2017).

## The bio‐based economy – facilitating current and future economic growth

As alluded to above, the creation of new employment opportunities is dependent on a number of parameters, such as demand, capital expenditure, distribution of the rents of productivity growth (UNCTAD, 2012), but balanced growth of existing commercial enterprises, for example through establishment of interconnected (specialized) local/regional R&D hubs (see e.g. Frost and Sullivan, 2011; Damond, 2014), and, importantly, the creation of innovative new businesses, are fundamental.

Numerous individuals and organizations have emphasized the economic potential of knowledge‐based business sectors, and the need to invest in and nurture them, and in the infrastructure needed for them to thrive. The bio‐based economy is one of the key sectors in this category, able to profit from the accelerating rate of new discoveries and technical developments. It is also one of the most investment‐ and human capital‐intensive, fastest growing, lucrative and flexible (e.g. scalable) innovation sectors. Globally, the bio‐based economy is projected to grow by at least 50% by 2030 (Bio, 2017). Whilst addressing local and regional challenges with biotech innovation, the bio‐based economy in low‐ and middle‐income countries can take advantage of the international pull effects for new solutions and thus emerging R&D opportunities. Currently, biotechnology is also one of the key drivers for medical innovation. Biosimilars, for example, represent one highly relevant area within the future biotech landscape (IMS Institute for Healthcare Informatics, 2016), as payer organizations in many healthcare systems, especially in high‐income countries, are seeking a broader array of, and less costly and more effective treatment alternatives. Provided adequate regulatory and quality measures are in place, public innovation funds are made available, and incentives for domestic and foreign direct investment are introduced, biotechnology will be an ideal and synergetic driver of the *demographic dividend* in a variety of settings, by offering opportunities to a growing and more highly skilled workforce, and increasing investment opportunities and human capital. It is ideally suited not only for R&D of new and more efficient products, processes and services, but also for diversification, that increase productivity and scale down waste: *less space, less matter, less waste, for more*.

### Microbial biotechnology – exceptional diversity of applications and opportunities for specialization

Microbial biotechnology is both one of the oldest technologies (fermentation history is as old as civilization) and one of the newest and most rapidly growing industries (the highest number of start‐ups, globally, are microbiome‐based biotechnology companies). The field has thus a long pedigree of experience and experimentation and is currently enjoying an exceptionally exciting period of continuous discoveries.

**Figure 1:**
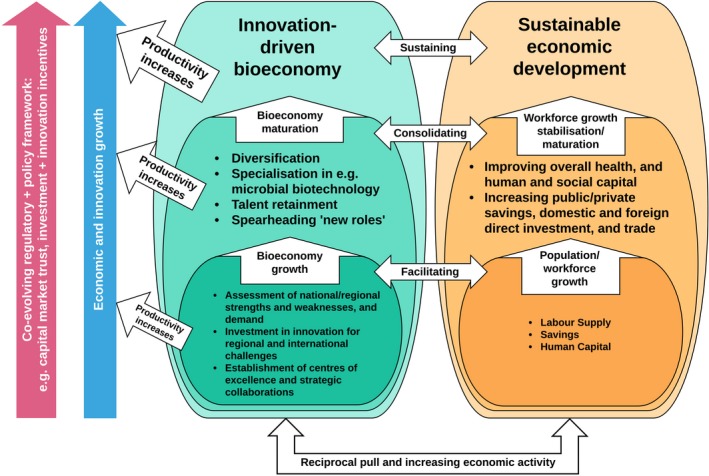
Growing the bioeconomy and increasing the demographic dividend to enhance economic development – illustration in part based on Bloom, Canning et al., 2003; McTaggart, Findlay etal., 2012.

Applications that resulted from or are now emerging out of advances in microbial biotechnology research are manifold and exceptionally diverse and include: diagnostics, pharmaceuticals, immune prophylactics, microbial therapies, fermented foods, food supplements, plant growth promotion and protection agents, biocatalysis and green chemistry, chemical feedstocks, biopolymers and biomaterials, biosurfactants and emulsifiers, compatible solutes and bioprotectants, fermentation, bioenergy from renewable feedstocks, fossil fuel recovery and valorization, bioelectricity, clean water provision, recycling technologies, wastewater treatment, bioremediation, biomining, metal and nutrient recovery, carbon capture, self‐healing concrete, protection of cultural goods. The diversity of applications is on the same scale as that enjoyed by chemistry and chemicals. And, importantly, the range of diversity of applications is expanding weekly, witness the explosion of new applications in the field of microbiome technology. This means that the range of possibilities for specialization and niche products from microbial biotechnology is exceptionally wide and can thus aliment many large, medium and small enterprises with profitable business opportunities (see also SDG *8.2 Achieve higher levels of economic productivity through diversification, technological upgrading and innovation, including through a focus on high‐value added and labour‐intensive sectors*; see e.g. United Nations, 2015a,b).

### Microbial biotechnology – exceptional innovation and entrepreneurial actors

Application diversity potential does not, of course, automatically translate into commercial activities, business expansion or growth in employment: the potential has to be mined, concretized, developed, refined and marketed, all of which require talented innovators working in a favourable environment. Innovation and entrepreneurial approaches are key in unlocking new products, processes and services, gaining market access, and successful commercialisation.

There are a number of parameters that can be taken into consideration for assessing the innovation level of a particular sector, including the annual number of patent applications/research papers published/scientific meetings, the rate of creation of new dedicated university departments, rate of creation of start‐ups, and by all these parameters, biotechnology is seen to be highly innovative. For a current snapshot of innovations in diverse sectors of microbial biotechnology, the reader is directed to the contributions published in this issue, particularly those by (Alvarez and Fernandez, 2017; Caselli, 2017; Cordero *et al*., 2017; Dürre, 2017; Felgner *et al*., 2017; Garcia, 2017; Godoy *et al*., 2017; Gurry, 2017; Lee, 2017; Li and Gadd, 2017; de Lorenzo, 2017; Macaskie and Orozco, 2017; Narancic and O'Connor, 2017; Nealson, 2017; Ramos, 2017; Schmidt‐Dannert, 2017; Sherry *et al*., 2017; Trivedi *et al*., 2017; Verstraete and Devrieze, 2017; Webster, 2017) – see also SDG *8.3 Promote development‐oriented policies that support productive activities, decent job creation, entrepreneurship, creativity and innovation, and encourage the formalization and growth of micro‐, small‐ and medium‐sized enterprises, including through access to financial services* (see e.g. United Nations, 2015a,b).

### Need to nurture innovation

While the long‐term success of an organization is determined by a number of indicators, originality and innovation are undoubtedly paramount, both for its successful evolution and for funder/customer perception of its quality and potential. Innovation itself is influenced by a number of factors, particularly the availability, density and diversity of innovators, newest information and ideas, the prevailing degree of ‘innovation culture’ and the influence of innovation facilitators, such as administrators and managers – gatekeepers – who decide on the further development of discoveries/advances. Innovation is often taken for granted/discussed as though it were a commodity – a parameter among many to be managed – but true innovators, like the best artists, surgeons, violinists, managers, politicians, are a rare species and require explicit support, which is frequently a necessary degree of ‘guided’ freedom, in order to flourish. Productive, synergistic innovator:gatekeeper partnerships are crucial, so gatekeepers need to be involved at an early stage in development of innovative ideas in order to provide the support needed for further development and commercialization.

### Sustainability considerations will shift perception of application value

While many biotechnology products and processes are inherently high value, others (particularly those that improve the quality of degraded environments, such as bioremediation and biomining) tend to be perceived as low value, often uncompetitive with existing feedstocks and technologies (frequently due to an unfavourable regulatory framework), and hence less attractive for development and investment. However, the recognition of the benefits of such products and processes, and hence their competitiveness, is fundamentally changing with integration of sustainability principles into economic analyses, their consideration not as stand‐alone but rather as components in cyclical processes and as participating elements in global issues (e.g. carbon footprints, energy vs. food, travel/food distribution and disease transmission control, food costs/food security/producer poverty, energy sourcing/energy security/political independence, land use and biodiversity; see the SDG texts in United Nations, 2015a,b), and resulting changes in the regulatory landscape. Thus, some currently low value and/or poorly competitive microbial technologies will in the future gain importance and attract investment as a result of changing perceptions, policies, regulatory frameworks and increasing benefit.

### The increasing importance of artificial intelligence for biotechnology – shifting specialization focus

For some time now, there has been a continual acceleration in the speed of data collection and the volumes collected (in the biotechnology sphere e.g. via next‐generation sequencing, high‐throughput assays). Artificial intelligence^1^ (AI) will become the primary means of handling and analysing these vast amounts of data, and thus a major driver of new discoveries across diverse scientific disciplines and industries. AI's continuously evolving sophistication, and thus ability to rapidly detect and ‘learn’ from patterns in vast data sets (numeric parameters, images, videos, etc., and, if appropriately equipped with sensory hardware, observe, probe and examine living beings and other phenomena), will revolutionize the efficiency, i.e. speed, accuracy and breadth, of data analysis. AI will be able to identify or predict previously unknown relationships of correlation, correlation direction, causality, etc. (see e.g. Shanahan, 2015), and costs for input, pattern detection and prediction tasks will drop significantly. It has therefore been suggested that the current *modus operandi* of research and development in public and private organizations will change dramatically. Roles for employees affected by these changes will increasingly shift towards appraisal and judgement of the patterns, predictions and inferences suggested by generic and specialized AI (Agrawal *et al*., 2016). A central function for these new roles of judgement will be to adequately investigate and understand the rationale (criteria, parameters, attributes, etc., i.e. data sets) according to which AIs have evolved and in which direction the AIs have become increasingly biased^2^ (their heuristics, hypotheses formulation, etc.), in order to judge the validity of AI‐based findings (see e.g. House of Commons Science and Technology Committee, 2016; Ingle *et al*., 2016; Witten *et al*., 2016; Bughin *et al*., 2017). Microbial biotechnology exploits big data in most of its diverse branches, so it will be a major beneficiary of AI‐driven acceleration of data mining, prediction and discovery. This means that AI will drive the creation of new enterprises and employment opportunities in the microbial biotechnology arena, but also changes in the roles of existing enterprises and employees. The sustainable and adequate exploitation of AI, including the proper scrutiny of AI‐findings according to scientific best practice, will necessitate significant investment in educational and collaboration programmes that specifically maximize expertise in judging and translating AI‐findings. This in turn will lead to improved research and development practices of microbial biotechnologies across small and large, public and private organizations.

## Investment in microbial biotechnology as a strategic priority for economic growth

### Enhancing bioeconomies and transnational value creation

Some of the authors of this paper proposed microbial biotechnology as a priority target for infrastructure investment for economic revival of Southern European countries, following the recent economic crisis that particularly affected these countries (and still does) (Timmis *et al*., 2014). The idea was: all of these countries have excellent education systems and research expertise, but relatively modestly funded research bases and little and/or fragmented biobusiness. They therefore have potent capacities to develop biobusiness but lack the necessary critical mass and political support. We argued that investment of structural funds to create this critical mass and new research networks would have a greater and more sustainable long‐term impact on economic recovery than traditional infrastructure investments, like highways and building programmes. While the investment concept for all countries was the same: building cutting edge research infrastructure, significant expansion of selective research capacity in regional hubs, creation of national networks for discovery of new chemicals through prospecting microbial diversity and the harnessing of cell biology groups for screening for agonists–antagonists of newly discovered cellular targets, the application sector of each would be different to reflect their distinct cultures, resources/access to resources and business/socio‐political priorities: e.g. Spain is strong in systems and synthetic biology, Portugal has strong cultural links and privileged access to biodiversity of former colonies, Italy is a leader in exploration of rare extreme deep sea brine lakes, Greece has a long tradition in maritime trade, and so forth. Even without the obvious distinctions of history, geography and culture, different regions have different microbial strains which constitute unique resources, e.g. for the local development, production and exploitation of locally derived inoculants for growth promotion and protection of crop plants in agriculture. Thus, each country could specialize in different biotechnology sectors – medical applications, agriculture, environment, and so on. SO: all can cooperate, share experiences and develop synergies one with another, because they do not need to directly compete in a significant manner. Crucially, the concept presented in the article proposes the creation of a professional integrated financial, business and infrastructure framework for the rapid creation of start‐ups by young investigators of the networks that make the new discoveries (Timmis *et al*., 2014).

Since the publication of this article, interest in the concept has been expressed from East European and Latin American countries, which have similar potential. Like the countries of Southern Europe, a number of countries in Latin American and Asia have outstanding medical systems which could serve as a major resource and crystallization factor for medical biotechnology biobusiness, developing and trialling new medical and nutritional products. Low‐ and middle‐income countries often have highly valuable biological resources that can be utilized in the development of new biotechnology applications. They can either develop these resources themselves, by investing in the necessary education, training, infrastructure and leadership, or by developing synergistic, mutually beneficial cooperations of stewardship and strategic joint commercial ventures with appropriate partners from other settings.

Thus, this proposal to support economic revival and enterprise and employment creation through investment in microbial biotechnology has universal applicability, given the appropriate political mindset and determination. And the creation of regional activities based on regional specificities creates regional stakeholders that encourage regional efforts and inclusiveness, corporate identity, commercial participation in regional society, including education, health, culture, and of course regional partnerships.

### Retaining the return on investment in education and recruiting new talent

A potential problem for countries with underinvestment in biotech R&D and business is that their aspirations may be frustrated by a *brain drain* – their best talent migrates and then fails to return – thereby constituting a *displaced educational investment* and a suboptimal use of national resources. Moreover, those countries to which talent migrates tend to have their own highly talented workforce, which is thereby reinforced and, as a consequence, the national *R&D gap* between such countries increases even further. However, in a highly diverse global economy, in which individual countries specialize in diverse activities in which they strategically invest to become and remain leaders in the field, highly trained individuals will be incentivized to migrate only temporarily and return to provide a return on investment. Therefore, selective investment in higher education and research training – together with establishment of the relevant specialist biobusiness – should be considered priorities to create a regional pool of talent, expertise and innovation that provides long‐term benefits to both the regional and global economy. In any case, *a diverse, highly motivated, talented, experienced and effectively networked bioeconomy workforce must be considered to be a key global economic, intellectual and cultural ‘good’ of our global society*.

### Attracting young professionals to the most dynamic sectors of biobusiness

While there are a number of media which inform about current recruitment for individual open positions, it is also important to provide more generic information about expanding business sectors that offer high employment potential. As an example of this, to encourage young biotechnology professionals to gravitate to rapidly evolving business sectors with high recruitment levels, or at least to be aware of the employment opportunities available, Microbial Biotechnology is currently publishing a series of Editorials on the opportunities of enterprise and employment creation generated by microbial technology. This series has so far featured Editorials on microbiome transplant therapy (van der Lelie *et al*., 2017), microbiome‐based products for agriculture (Singh, 2017), microbiome‐based human forensics (Hampton‐Marcell *et al*., 2017), do‐it‐yourself technologies as sources of innovation in microbial biotechnology (de Lorenzo and Schmidt, 2017), bioprospecting unusual environments (Tanner *et al*., 2017) and non‐antibiotic feed adjuncts for food animals (Brüssow, 2017). Others are in the pipeline.

## Conclusions

Microbial biotechnology is a key knowledge‐based biobusiness that has applications in almost all aspects of life and society and will be a major driver of enterprise and employment creation in the future, and hence significantly contribute to SDG 8: Promote sustained, inclusive and sustainable economic growth, full and productive employment and decent work for all. Importantly, microbial biotechnology offers manifold opportunities for the creation and exploitation of diverse partnerships providing more than mere commercial benefits. Implementation of top‐down measures for fulfilment of the SDGs – together with bottom‐up growing societal demands – will foster new areas of biotechnological activity in both developed and developing countries. Frontline research will thereby reach out to topics and scientific territories typically away from the radar of investors e.g. large‐scale CO2 capture, development of new antibiotics, combatting poverty‐associated diseases, and multi‐scale environmental rehabilitation. The drivers of this expansion will include not just the habitual hypertechnological actors that have traditionally dominated the realm of the bio‐based economy, but also *frugal* technologists able to translate conceptually sophisticated approaches into user‐friendly and affordable platforms. This will certainly help not only to democratize the functioning of the biotechnological market, but also enable ideally all countries and communities to access the type of sustainable prosperity that only modern biosciences, and in particular microbial biotechnology, can achieve.

However, realization of the potential of microbial biotechnology to facilitate attainment of SDG 8 needs support from policy makers, business and educators who need to recognize that its fundamental strengths – high innovation, local and regional innovation potential and diversity of applications – must be fostered/supported by both investment and gatekeepers. Key factors to maximize the utility of microbial technology are:

- Exploit local and historical strengths and resources (e.g. Brazil and bioethanol) to increase global diversification and specialization, and facilitate the formation and maintenance of strategic collaborations and partnerships, provide an environment (higher education, research) in which strategic research and know‐how become and remain among the best in the world, and innovation thrives, and ensure key groups stay at the cutting edge in selected strategic fields.
- Establish and finance strong strategic links between academia and industry to accelerate commercialization; facilitate start‐ups, acquire and support missing skills, infrastructure and involve gatekeepers as early as possible in the process of innovation so that they can more effectively support progress.
- Establish a policy, regulatory and economic environment favourable to bioeconomy development and success. Make innovation a key strategic component of infrastructure investment and enact policies that favour and support entrepreneurs.
- In more general terms, when considering investment options to increase/facilitate economic growth, always identify and quantify the *rate limiting parameter* and then decide which option would best alleviate it (will this new construction project bring significant benefits over a 5‐year/10‐year/20‐year time span? If so, how does the calculated benefit compare with that of investment in an innovation sector?).

Microbial biotechnology is significantly contributing, and has exceptional potential to contribute much more, in diverse arenas, directly to 10 of the 17 SDGs, one of which, SDG 8, is the subject of this article, and the others of which are discussed in this issue. Moreover, progress towards SDG 8 will also necessarily impact positively on other SDGs, in particular SDGs 1^3^ and 10,^4^ but also 4,^5^ in as much as embracing and promoting high‐tech, innovative biobusiness necessarily requires high levels of education for a growing workforce, SDG 5,^6^ as full employment is *inter alia* facilitated by and re‐inforces gender equality and empowerment, and SDGs 16^7^ and 17,^8^ in so far as high‐tech industries need highly educated work forces which usually engender societies favouring peace, inclusiveness and sustainability. Thus, microbial biotechnology contributes/will contribute, directly or indirectly, to almost all of the SDGs.

## Conflict of interest

None declared.

## Notes

1 We use artificial intelligence here as an umbrella term for the broad field of ‘self‐learning’ algorithms and applications, such as supervised and unsupervised machine‐learning, convolutional neural networks.

2 For a more detailed account of different biases, such as the language, search and overfitting‐avoidance bias, in *inter alia* machine learning, see e.g. (Witten *et al*., 2016).

3 SDG1: *End poverty in all its forms*.

4 SDG 10: *Reduce inequality within and among countries*.

5 SDG 4: *Ensure inclusive and equitable quality education and promote lifelong learning opportunities for all*.

6 SDG 5: *Achieve gender equality and empower all women and girls*.

7 SDG 16: *Promote peaceful and inclusive societies for sustainable development, provide access to justice for all and build effective, accountable and inclusive institutions at all levels*.

8 SDG 17: *Strengthen the means of implementation and revitalize the global partnership for sustainable development*.
